# A Comparison of Three Quantitative Methods to Estimate G6PD Activity in the Chittagong Hill Tracts, Bangladesh

**DOI:** 10.1371/journal.pone.0169930

**Published:** 2017-01-25

**Authors:** Benedikt Ley, Mohammad Shafiul Alam, James J. O’Donnell, Mohammad Sharif Hossain, Mohammad Golam Kibria, Nusrat Jahan, Wasif A. Khan, Kamala Thriemer, Mark D. Chatfield, Ric N. Price, Jack S. Richards

**Affiliations:** 1 Global and Tropical Health Division, Menzies School of Health Research and Charles Darwin University, Darwin, Australia; 2 Infectious Diseases Division, International Centre for Diarrheal Diseases Research, Bangladesh, Mohakhali, Dhaka, Bangladesh; 3 Centre for Biomedical Research, Burnet Institute, Melbourne, Victoria, Australia; 4 Centre for Tropical Medicine and Global Health, Nuffield Department of Clinical Medicine, University of Oxford, Oxford, United Kingdom; 5 Department of Medicine, University of Melbourne, Parkville, Victoria, Australia; 6 Victorian Infectious Diseases Service, Peter Doherty Institute for Infection and Immunity, Melbourne, Victoria, Australia; Institut national de la santé et de la recherche médicale - Institut Cochin, FRANCE

## Abstract

**Background:**

Glucose-6-phosphate-dehydrogenase-deficiency (G6PDd) is a major risk factor for primaquine-induced haemolysis. There is a need for improved point-of-care and laboratory-based G6PD diagnostics to unsure safe use of primaquine.

**Methods:**

G6PD activities of participants in a cross-sectional survey in Bangladesh were assessed using two novel quantitative assays, the modified WST-8 test and the CareStart^™^ G6PD Biosensor (Access Bio), The results were compared with a gold standard UV spectrophotometry assay (Randox). The handheld CareStart^™^ Hb instrument (Access Bio) is designed to be a companion instrument to the CareStart^™^ G6PD biosensor, and its performance was compared to the well-validated HemoCue^™^ method. All quantitative G6PD results were normalized with the HemoCue^™^ result.

**Results:**

A total of 1002 individuals were enrolled. The adjusted male median (AMM) derived by spectrophotometry was 7.03 U/g Hb (interquartile range (IQR): 5.38–8.69), by WST-8 was 7.03 U/g Hb (IQR: 5.22–8.16) and by Biosensor was 8.61 U/g Hb (IQR: 6.71–10.08). The AMM between spectrophotometry and WST-8 did not differ (p = 1.0) but differed significantly between spectrophotometry and Biosensor (p<0.01). Both, WST-8 and Biosensor were correlated with spectrophotometry (r_s_ = 0.5 and r_s_ = 0.4, both p<0.001). The mean difference in G6PD activity was -0.12 U/g Hb (95% limit of agreement (95% LoA): -5.45 to 5.20) between spectrophotometry and WST-8 and -1.74U/g Hb (95% LoA: -7.63 to 4.23) between spectrophotometry and Biosensor. The WST-8 identified 55.1% (49/89) and the Biosensor 19.1% (17/89) of individuals with G6PD activity <30% by spectrophotometry. Areas under the ROC curve did not differ significantly for the WST-8 and Biosensor irrespective of the cut-off activity applied (all p>0.05). Sensitivity and specificity for detecting G6PD activity <30% was 0.55 (95% confidence interval (95%CI): 0.44–0.66) and 0.98 (95%CI: 0.97–0.99) respectively for the WST-8 and 0.19 (95%CI: 0.12–0.29) and 0.99 (95%CI: 0.98–0.99) respectively for the Biosensor. Hb concentrations measured by HemoCue^™^ and CareStart^™^ Hb were strongly correlated (r_s_ = 0.8, p<0.001, mean difference = 0.09 g Hb/dL, 95% LoA: -2.15 to 2.34).

**Conclusion:**

WST-8 and the CareStart^™^ G6PD Biosensor represent advances in G6PD diagnostics in resource poor settings, but will require further development before clinical deployment. The CareStart^™^ Hb instrument produced a precise measure of haemoglobin concentration.

## Introduction

Glucose–6–Phosphate Dehydrogenase (G6PD) is the rate limiting enzyme in the pentose phosphate pathway that protects human cells from oxidative stress. G6PD generates the reduced form of nicotinamide adenine dinucleotide phosphate (NADPH) which in turn maintains sufficiently high levels of reduced glutathione, essential to bind free radicals and protect human cells from the adverse effects of oxidative stress [1]. Red blood cells (RBCs) are particularly vulnerable to oxidative stress and in the absence of a nucleus depend on the G6PD enzyme generated during erythropoiesis. The half-life of RBCs with lower G6PD activity is significantly shortened as the tolerance towards oxidative stress is reduced [1].

G6PD deficiency (G6PDd) is one of the most common enzymopathies in humans, affecting an estimated 400 million individuals worldwide [2]. It is an X-linked enzyme deficiency; men are either hemizygous G6PD deficient or normal, whilst women can be homozygous normal or deficient or heterozygous deficient. In the latter, enzyme activity varies with the degree of lyonization [1, 3]. To date more than 185 clinical variants of the G6PD gene have been reported, associated with a wide spectrum of enzyme activity [4, 5].

Primaquine (PQ), an 8-aminoquinoline (8-AQ), is the only drug currently available with activity against the dormant liver stages of *P*. *vivax* (hypnozoites), but the drug can induce oxidative stress and cause severe haemolysis in individuals with G6PDd [6]. The most widely used diagnostic test for G6PDd is the fluorescent spot test (FST), a qualitative assay that requires basic laboratory infrastructure [7, 8]. The gold standard for G6PDd diagnosis is UV spectrophotometry which quantifies G6PD activity, and flow cytometry to determine the degree of lyonization in females [3, 9].

Over the last few years a number of G6PD diagnostic assays have been introduced to the market [10], aimed at facilitating bedside point of care diagnosis. However all of the devices suitable for field applications are limited to a qualitative result based on a G6PD cut–off of approximately 30% enzyme activity, the cut-off activity most widely used to guide whether primaquine based radical cure should be administered or not [3]. These qualitative tests do not identify reliably individuals with intermediate G6PD activity (30–60%), such as heterozygous females [10]. Access Bio^®^ recently introduced the CareStart^™^ G6PD Biosensor, a handheld digital device which uses venous or capillary blood in single-use strips to quantify G6PD activity at the bed side in 4 minutes. Quantification of G6PD enzyme activity for all tests requires normalisation of the results against either red blood cell count or haemoglobin (Hb) concentration. Hence the Biosensor is marketed with a complementary handheld device for measuring Hb concentration (CareStart^™^ Hb).

An alternative quantitative assay has recently been developed based on the WST-8 test, which has been further adapted to give a rapid, cheap test amenable to batch testing for population studies using a 96 well microplate and read by an ELISA plate reader [11, 12].

The aim of this study was to assess the utility and performance of the CareStart^™^ G6PD Biosensor, the WST-8 G6PD test and the Carestart^™^ Hb machine.

## Methods

### Study design, site and period

A cross-sectional survey was conducted between August 2015 and January 2016 in the Chittagong Hill Tracts (CHT), Bangladesh. In this area both *P*. *falciparum* and *P*. *vivax* are endemic with a prevalence of 67.6% and 32.4% respectively found in a hospital based survey in 2014–2015 [13]. The CHT are the least densely populated area of Bangladesh with a mixed population of Tibeto—Asian and Bengali descent [14]. In this area the prevalence of G6PD activity <30% is 6.4%, prevalence of G6PD activities between 30% to <60% is 35% [15].

### Participant enrolment

Villages were purposively selected based on accessibility. Households within selected villages were selected at random and one person per household randomly selected to avoid an artificial dominance of specific G6PD gene-variants.

### Study procedures

After obtaining written informed consent a maximum of 3ml venous EDTA blood as well as capillary blood from a finger prick were collected.

#### HemoCue^™^ and CareStart^™^ Hb machine

A cuvette was inserted into the HemoCue^™^ (Hb201+, Angelholm, Sweden) device and the tip of the cuvette held directly against a drop of blood from the finger prick. For the CareStart^™^ Hb test machine (MHD-1, Access Bio, USA) a drop of capillary blood was collected with a micropipette provided with the device and placed on a single use strip inserted into the machine. Hb levels measured by both methods were recorded.

#### G6PD Biosensor

Each day prior to testing, the reliability of the G6PD Biosensor was assessed by using a control strip. A box specific chip was provided with every box of strips that enabled the Biosensor to recognize the lot number of the single use strip. For every new lot of strips the corresponding chip was inserted into the machine for that entire batch.

The G6PD Biosensor was undertaken on capillary blood in the field. For each test a single use strip was applied and the displayed result recorded. If the G6PD Biosensor returned an error message during sample testing, the error message was recorded, the problem addressed whenever possible and the measurement repeated. If the second measurement also returned an error message, the message was recorded, but no further samples were taken (S1 File).

#### Spectrophotometer

Following venepuncture, blood was stored immediately at 4–8°C, transported to a reference laboratory at the International Centre for Diarrheal Disease Research, Bangladesh (icddr,b) in Dhaka, and processed within 7 days. Spectrophotometry was conducted using kits from Randox^®^ (UK) on a Shimadzu 1800^™^ (Kyoto, Japan) spectrophotometer. Normal and deficient G6PD controls comprised of lyophilised hemolysates (Randox^®^, UK) were run with every test run and results were only considered valid if the measured activities of the controls were within the recommended reference range. Enzyme activity was calculated and adjusted for the Hb concentration using the HemoCue^™^ value recorded at the time of sample collection to create the standard unit of G6PD enzyme activity; units of G6PD activity per gram of Hb (U/g Hb).

#### WST-8 G6PD measurement

The WST-8 G6PD test was performed on the venous blood sample within 7 days of sample collection in a 96 well microplate at the reference laboratory in Dhaka, according to established methods (O’Donnell *et al*-in preparation). The colour intensity of the WST-8 formazan product was quantified as an end-point value after 15 minutes incubation using a microplate reader (BioTek ELx808, USA) and converted into a quantitative G6PD enzyme activity result, after adjusting for the Hb concentration.

### Statistical analysis

All data were entered in Epidata (Denmark) and analysed using Stata version 14 (Stata Corp, USA). Hb concentrations measured by the HemoCue^™^ were used to calculate U/g Hb for all G6PD tests. The adjusted male median (AMM) G6PD activity (100% G6PD activity) was calculated for each G6PD assay [16]. The AMM was defined as the median G6PD activity of all male participants after excluding samples with less than 10% of the overall median activity. Severe, moderate and mild G6PD deficiency were defined as <10%, 10-<30% and 30-<60% G6PD activity of the AMM. Any activity ≥60% of the AMM was defined as G6PD normal (adapted from WHO definitions [17]).

Mean G6PD activity and Hb concentration were compared using the Wilcoxon matched-pairs signed-ranks test, Spearman’s rank correlation (r_s_) and Kappa statistics. A Bland-Altman plot analysis was used for the direct comparison between different tests. Categorical data were compared using McNemar’s test for correlated proportions. In order to compare overall performance of the tests at specific G6PD enzymatic activity cut—offs (i.e. <10%, 10–30%, 30–60% and ≥60%), the areas under the ROC curve were compared.

Sensitivity, specificity and predictive values were calculated for different threshold activities applying standard formulas [18, 19]. Samples with enzyme activities below a defined fraction of the AMM were defined as positive results (G6PD deficient), those that had a higher activity were defined as negative results (G6PD normal). To determine test performance at a specific fraction of the AMM, the corresponding G6PD activity for each assay was calculated. A sample with a measured G6PD activity below the calculated threshold activity for the reference and the test assay was defined as a true positive sample. True negative, false positive and false negative results were defined accordingly.

### Ethics

The study was approved by the ethical review committee (ERC), the research review committee (RRC) of the icddr,b (PR-15021) and the Human Research Ethics Committee (HREC) of the Northern Territory, Australia (HREC 2015–2336). Written informed consent was collected from all participants prior to enrolment, in case of minors written informed consent was collected from a legal guardian, if the minor was above the age of 11 years written informed assent was in addition collected from the minor.

## Results

Between 22^nd^ August 2015 and 11^th^ January 2016 a total of 1002 participants were enrolled, of whom the majority (n = 600, 60%) were females. The median age was 31 years (interquartile range (IQR): 17–45 years, range: 5–80 years). G6PD status was determined in 999 (99.7%) participants by spectrophotometry and in 997 (99.5%) by CareStart^™^ G6PD Biosensor and the WST-8 test, haemoglobin concentration was quantified by HemoCue^™^ and CareStart^™^ tests in all participants. On site testing was done immediately, but there was a median delay between sample collection and G6PD measurement of 2 days (IQR: 1–4) for spectrophotometry, and 4 days (IQR: 3–5) for the WST-8 assay (p<0.001). Correlation between time to measurement and G6PD activity was very small for spectrophotometry (r_s_: -0.13, p<0.001) and not significant for the WST (r_s_: 0.03, p = 0.4).

### Measurement of G6PD activity

**Fig 1:**
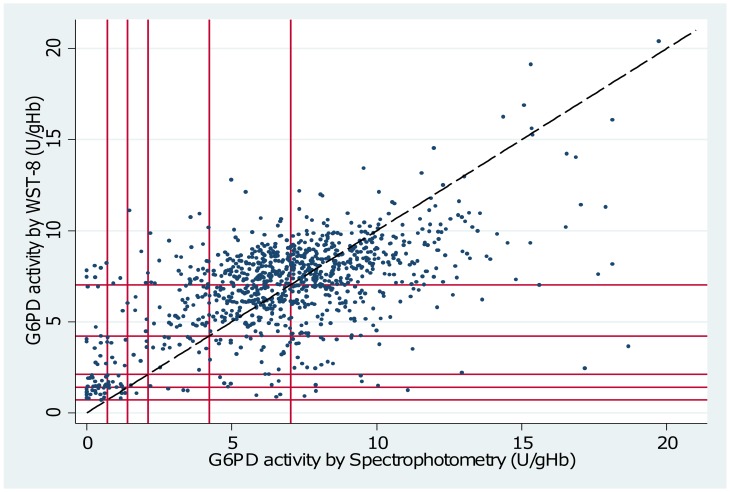
Scatter plot of G6PD activity measured by Spectrophotometry vs. WST-8. Diagonal line = line of equality. Starting from origin outwards vertical and horizontal lines mark 10%, 20%, 30%, 60% and 100% activity of the adjusted male median based on spectrophotometry and WST-8 respectively.

**Fig 2:**
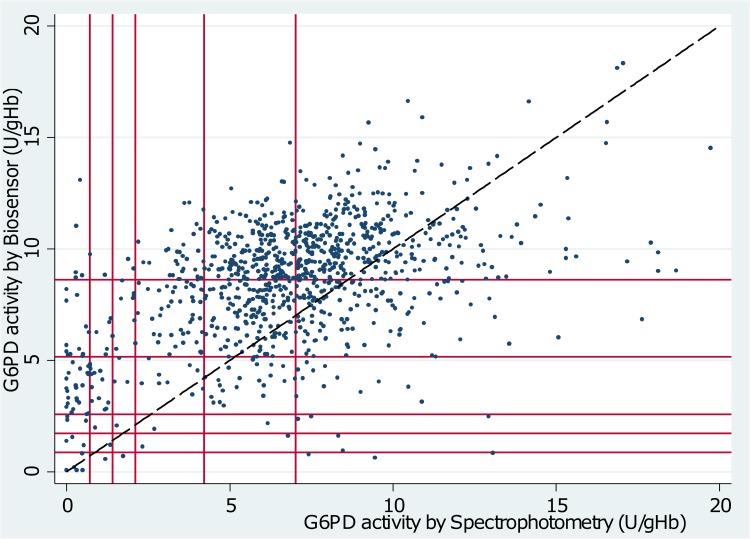
Scatter plot of G6PD activity measured by Spectrophotometry vs. Biosensor. Diagonal line = line of equality. Starting from origin outwards vertical and horizontal lines mark 10%, 20%, 30%, 60% and 100% activity of the adjusted male median based on spectrophotometry and Biosensor respectively.

**Fig 3:**
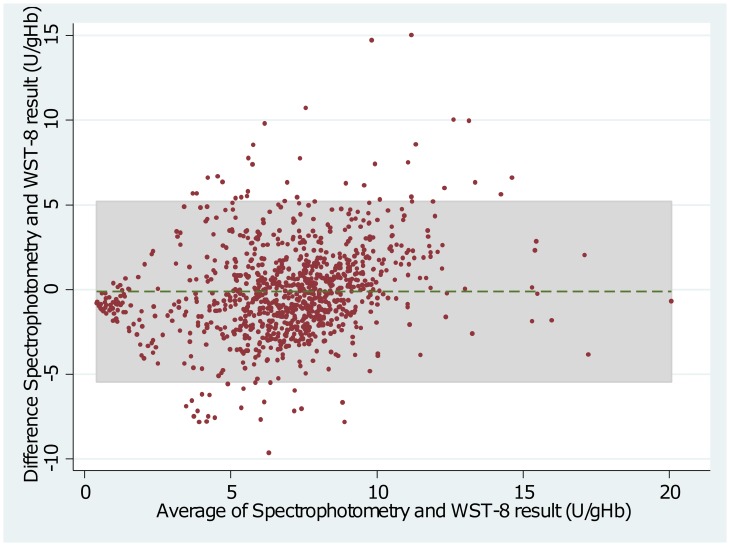
Bland—Altman Plot of Spectrophotometry and WST-8. Dotted line indicates mean difference, grey shaded area indicates 95% limits of agreement.

**Fig 4:**
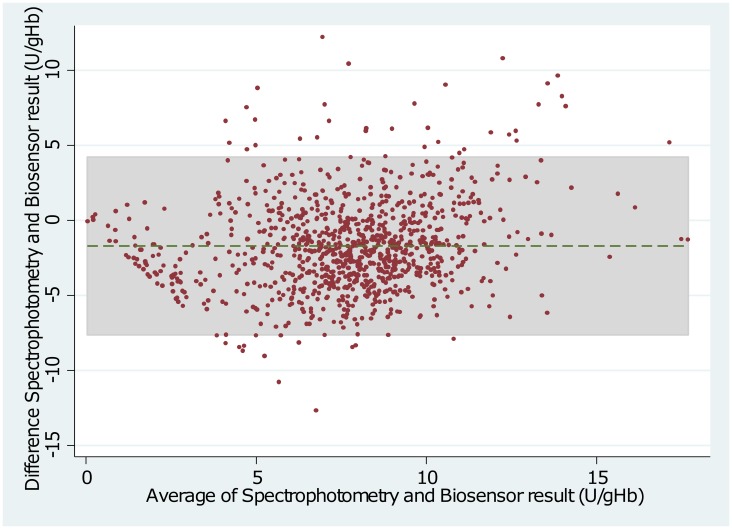
Bland—Altman Plot of Spectrophotometry and Biosensor. Dotted line indicates mean difference, grey shaded area indicates 95% limits of agreement.

The Adjusted Male Median (AMM) enzyme activity was 7.03 U/g Hb (interquartile range (IQR): 5.38 to 8.69) when measured by spectrophotometry compared to 7.03 U/g Hb (IQR: 5.22 to 8.16) by WST-8 (p = 1.00) and 8.61 U/g Hb (IQR: 6.71 to 10.08) by Biosensor (p<0.01)(see also Figs 1 and 2). The mean difference between spectrophotometry and WST-8 was -0.12 U/g Hb (95% limit of agreement (95% LoA): -5.45 to 5.20), and between spectrophotometry and the Biosensor was -1.74 U/g Hb (95% LoA: -7.63 to 4.23) (Figs 3 and 4). Correlation between spectrophotometry and WST-8 measurement was slightly stronger (r_s_ = 0.51) than that between spectrophotometry and Biosensor measurements (r_s_ = 0.44), (Table 1).

**Table 1:** Results of correlation and Bland Altman plot.

Comparison	n	Spearman’s Rank Correlation (r_s_)	Mean differenceU/g Hb (G6PD activity) or g/dL (Hb)	95% limits of agreement
Spectrophotometry vs. Biosensor	994	0.44	-1.70	-7.63 to 4.23
Spectrophotometry vs. WST-8	995	0.51	-0.12	-5.45 to 5.20
HemoCue vs. CareStart Hb	1002	0.80	0.09	-2.15 to 2.34

**Fig 5:**
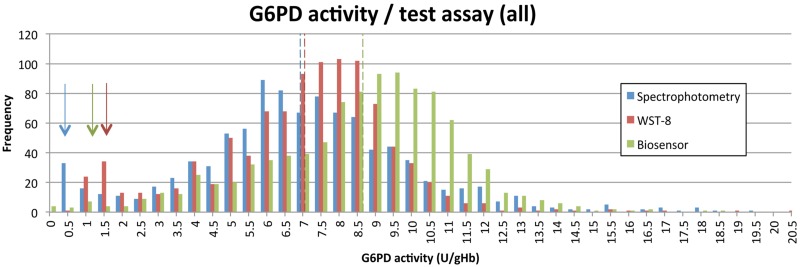
G6PD activity distribution according to different assays among the entire study population. Arrows indicate the peak for G6PDd samples / assay, dotted lines indicate AMM / assay.

**Fig 6:**
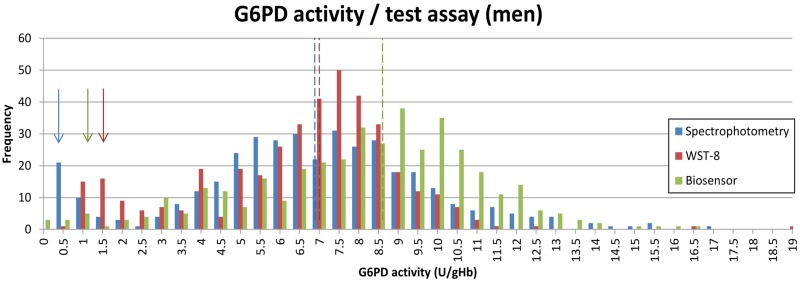
G6PD activity distribution according to different assays among the male study population. Arrows indicate peak for G6PDd samples / assay, dotted lines indicate AMM / assay.

The distribution of G6PD activities as measured by spectrophotometry and the WST-8 showed two distinct peaks for G6PD normal and G6PDd participants, while this was far less apparent for results derived from the Biosensor (Figs 5 and 6).

Both the WST-8 and the Biosensor identified significantly less truly G6PDd participants with G6PD activities below 10% and 30% (all p<0.05) compared to spectrophotometry (Table 2). When comparing the areas under the ROC curves between the WST-8 and Biosensor no significant difference (all p>0.05) was observed irrespective of cut-off activity applied (≤10%, ≤30% and ≤60% as derived by spectrophotometry). Areas under the ROC curve were largest at 30% cut-off activity as determined by spectrophotometry for both, the WST-8 (0.89, 95%CI: 0.84–0.93) and the Biosensor (0.88, 95%CI: 0.84–0.92).

**Table 2:** Category matches, Spectrophotometry vs. WST-8 and Spectrophotometry vs. Biosensor.

		**WST-8**					**Biosensor**				
	**G6PD activity**	<10%	10-<30%	30-<60%	> = 60	Total	<10%	10-<30%	30-<60%	> = 60	Total
**Spectrophotometry**	<10%	0 (0.0%)	31 (3.1%)	9 (0.9%)	9 (0.9%)	49	5 (0.5%)	7 (0.7%)	21 (2.1%)	17 (1.7%)	50
	10%-<30%	0 (0.0%)	18 (1.8%)	11 (1.1%)	11 (1.1%)	40	2 (0.2%)	3 (0.3%)	17 (1.7%)	17 (1.7%)	39
	30%-<60%	0 (0.0%)	4 (0.4%)	14 (1.4%)	66 (6.6%)	84	0 (0.0%)	2 (0.2%)	9 (0.9%)	73 (7.3%)	84
	> = 60%	0 (0.0%)	16 (1.6%)	42 (4.2%)	764 (76.8%)	822	3 (0.3%)	7 (0.7%)	37 (3.8%)	774 (78.5%)	821
	Total	0	69	76	850	995	10	19	84	881	994

The AMM is calculated for each assay and results are categorized and displayed based on the categories samples fall into / test assay applied.

Sensitivity, specificity, positive and negative predictive value of the WST-8 at 30% cut off activity were 0.55 (95%CI: 0.44–0.66), 0.98 (95%CI: 0.97–0.99), 0.71 (95%CI: 0.59–0.81) and 0.96 (95%CI: 0.94–0.97) respectively, the kappa statistic showed a moderate agreement of 0.59 (95%CI: 0.49–0.68).

At the same cut-off activity the Biosensor had a sensitivity, specificity, positive and negative predictive value of 0.19 (95%CI): 0.12–0.29), 0.99 (95%CI: 0.98–0.99), 0.59 (95%CI: 0.39–0.76) and 0.93 (95%CI: 0.91–0.94) respectively. Agreement between Biosensor and spectrophotometry was modest (Kappa = 0.26, 95%CI: 0.15–0.36).

### Measurement of Haemoglobin Concentration

The Hb concentration derived from the CareStart^™^ Hb machine correlated closely with that from the HemoCue^™^ (r_s_ = 0.8, p<0.001), with a mean difference of 0.09 g/dL (95% LoA: -2.15 to 2.34), (Table 1).

## Discussion

We assessed the utility of two novel assays for determining G6PD deficiency in a remote rural setting. Compared to the gold standard spectrophotometry, the WST-8 assay performed reasonably well and correlated with the results from spectrophotometry but had low sensitivity in identifying G6PDd individuals. The CareStart^™^ G6PD Biosensor was less well correlated with the gold standard, and a poor predictor of G6PD deficiency.

Males are either hemizygous G6PD deficient or normal, corresponding G6PD activities are therefore predicted to produce two distinct peaks in enzyme activity. Hence an indirect measure of a G6PD assay’s quality and robustness in field conditions is its ability to produce a low proportion of males with intermediate G6PD activity. In our study the gold standard spectrophotometry generated a distribution with two distinct peaks separating G6PD deficient and normal males. The WST-8 produced a peak for G6PDd males at a higher G6PD activity and the proportion of G6PD intermediate males was higher compared to spectrophotometry, whereas the Biosensor did not produce two clearly defined peaks (Fig 6).

The WST-8 is a quantitative assay designed as an alternative to the gold-standard spectrophotometry. Whereas spectrophotometry has considerable equipment and running costs, the WST-8 has modest equipment costs for a plate reader and incubator and costs only 0.10 USD per test. The mean difference between spectrophotometry and the WST-8 results was less than 2% of the AMM, which is unlikely to be of clinical significance. At a cut–off of 30% G6PD activity the WST-8 assay in this study had a sensitivity of 55% (49/89), resulting in a 6.9% (69/995) G6PDd prevalence in the study population compared to a prevalence of 8.9% (89/995) based on spectrophotometry. However the WST-8 assay did not identify any of the study participants with a G6PD activity less than 10%, a subgroup at greatest risk of severe primaquine induced haemolysis.

The Biosensor is a handheld device returning a quantitative result on G6PD activity within 4 minutes. The device costs approximately 500 USD and 2.50 USD per strip and is designed to guide treatment decisions at the point of care. As such the priority is to have a reliable diagnostic that can ensure patient safety by minimising exposure of vulnerable individuals to potential drug-induced haemolysis. At a 9% prevalence of G6PD deficiency, 7.5% (72/965) of individuals with a normal Biosensor result were actually severe or moderate deficient (<30% activity), 52.8% (38/72) of those were severe deficient (<10% activity) according to spectrophotometry. Overall only 19% (17/89) of severe or moderate deficient individuals (<30% G6PD activity) would have been identified by the Biosensor.

The poor performance of the two novel tests may reflect a number of limitations in our study design. The G6PD Biosensor was measured from capillary blood, whereas the WST-8 and spectrophotometry were performed on venous blood. Hb concentration, red blood and white cell counts have been shown to differ between capillary and venous blood and these can affect G6PD activity [20]. However a previous study in Thailand showed that none of these differences had a significant impact on measured G6PD activity [21]. Delay or faulty storage conditions could also have confounded our results. Whereas the Biosensor was used immediately at point of care, spectrophotometry and WST-8 required transport to a reference centre with a delay in laboratory processing of between 2 and 4 days. Thus G6PD activity could have fallen between collection and the time the samples were tested, indeed the enzyme activity was almost 19% lower by spectrophotometry than by the point of care Biosensor. However recent evidence suggests that G6PD activity remains stable for up to 21 days when samples are stored correctly [22], significantly greater than the time taken to process the samples in this study. Although the time between sample collection and spectrophotometry was negatively correlated with G6PD activity (r_s_ = -0.13), the relationship was too small to explain the observed differences (S1 Fig). There was no respective correlation for the WST-8 test. Another possible explanation for the discordant results is that the gold-standard UV spectroscopy assay itself may have performed suboptimally. The particular UV spectroscopy assay used in this study is technically complex requiring several erythrocyte wash steps prior to digitonin lysis and then use of the supernatant within 2 hours to measure G6PD activity. Each sample took approximately 45–60 minutes to process and read the G6PD activity, which created a significant workload on the laboratory staff. Further comparative studies of different UV spectroscopy assays are also needed and further indicate the need to develop simpler, cheaper quantitative G6PD tests for high-throughout testing in population studies.

The interpretation of G6PD activity requires adjustment for variation in Hb concentration or red cell mass. In parallel to the G6PD test assessments we also examined the utility of the CareStart^™^ Hb device compared to the widely used portable HemoCue^™^ test. The derived Hb measures showed excellent correlation, with a mean difference in readings of less than 0.1 g/dL. Considering the low cost of the device (~40 USD) and 0.30 USD per strip, the device is a good alternative to comparable formats.

Despite the suboptimal performance of both assays, the WST-8 and G6PD Biosensor represent significant advances for G6PD diagnostics, which with further development may be suitable for deployment in resource poor settings and with little laboratory infrastructure. An ELISA based quantitative assay such as the WST-8 has the potential to replace the costly and more complicated gold standard spectrophotometry, and thus the current format is being revised and developed with a complementary ELISA based Hb measurement method. The use of the Biosensor is amenable to deployment by healthcare workers within a couple of hours of training. A next generation of the Biosensor is likely to include an Hb measurement, thereby eliminating a further source of error. As momentum gathers to achieve the elimination of *P*. *vivax*, such robust field adapted methods will be critical for the widespread use of radical cure with an 8-aminoquinoline antimalarial.

## Supporting Information

S1 File Error messages returned by the Biosensor. (DOCX)

S2 File Underlying database. (XLS)

S1 Fig Spectrophotometry: G6PD activity / time to testing. (TIF)
