# Supplementary material for: A Comparison of Three Quantitative Methods to Estimate G6PD Activity in the Chittagong Hill Tracts, Bangladesh
Source: PLoS One. 2017 Jan 25;12(1):e0169930. doi: 10.1371/journal.pone.0169930 (PMC5266301; doi:10.1371/journal.pone.0169930)
Supplement: S1 File — (DOCX) [file pone.0169930.s001.docx]

**S1 File**

The Biosensor returned an error message in 1.4% (n=14) of all cases. In two cases a second attempt resulted in another error message, in 11 cases a valid result was returned on the second attempt and no second attempt was made in 1 case. In 6 cases the machine provided the error message that the ambient temperature was outside the machines range (error 1), when retesting was performed in a cooler setting the machine provided an acceptable result. In 8 cases the machine falsely assumed that the inserted strip had already been used (error 2), the same error message was returned during the second measurement in 2 cases.
